# Inhibitory effects of patchouli alcohol on hepatocellular carcinoma growth through accumulation of oxidative stress and inactivation of androgen receptor signaling

**DOI:** 10.7150/ijms.123787

**Published:** 2026-02-18

**Authors:** Xiao-Fan Huang, Kai-Fu Chang, Nu-Man Tsai

**Affiliations:** 1 Department of Medical Laboratory and Biotechnology, Chung Shan Medical University, Taichung 40201, Taiwan, R.O.C.; 2 Clinical Laboratory, Chung Shan Medical University Hospital, Taichung 40201, Taiwan, R.O.C.; 3 Department of Life-and-Death Studies, Nanhua University, Chiayi 62249, Taiwan, R.O.C.

**Keywords:** hepatocellular carcinoma, patchouli alcohol, sorafenib, androgen receptor

## Abstract

Hepatocellular carcinoma (HCC) is one of the most frequently diagnosed malignancies and exhibits a high mortality rate. Patchouli alcohol (PA) is a tricyclic sesquiterpene derived from *Pogostemon cablin*, and the present study evaluated the antihepatoma capacity of PA and described a potential strategy for its combination with sorafenib (SOR) *in vitro* and *in vivo*. The anticancer potential of PA against HCC was evaluated using the MTT assay, flow cytometry, western blotting, DCF-DA and JC-1 staining, TUNEL assay, immunofluorescence and immunohistochemistry staining, and migration and invasion assays. The results indicated that PA suppressed HCC growth by inducing reactive oxygen species (ROS) generation, mitochondrial membrane potential imbalance, and DNA damage, ultimately resulting in cell cycle arrest and apoptosis via the activation of p53/p21 and also extrinsic (Fas/FasL/caspase-8), intrinsic (Bax/Bcl2/caspase-9), and caspase-independent pathways. The combination of PA with SOR exhibited synergistic effects, exerted survival benefits, and improved the lifespan of mice at well-tolerated doses. Furthermore, PA targets the androgen receptor (AR) to inhibit dihydrotestosterone-induced (DHT)-induced cell proliferation, AR translocation to the nucleus, and downstream gene expression during HCC growth. On the whole, PA alone or in combination with SOR exhibited markedly improved therapeutic efficacy in HCC by blocking AR-mediated and multiple other signaling pathways. Therefore, this study provides an experimental basis for the evaluation of PA as an alternative drug (alone or in combination) for the treatment of HCC.

## Introduction

Liver cancer is an aggressive disease with poor prognosis 1, 2. According to estimates by the World Health Organization (WHO) in 2020, primary liver cancer was the sixth most common cancer and the third leading cause of cancer death with 905,677 new cases and 830,180 deaths, and this included hepatocellular carcinoma (HCC; 75%-85%), intrahepatic cholangiocarcinoma (10%-15%), and other rare types 3. Currently, surgery remains an effective approach to improve survival benefits for patients with HCC at an early stage; however, the high recurrence rate reaches 40% within 4 years and affects the therapeutic efficacy for HCC 4. Chemotherapy is one of the most important treatments for advanced HCC and can be classified as systemic chemotherapy for patients with extrahepatic metastases and as hepatic arterial infusion chemotherapy (HAIC) for patients with localized advanced HCC. Sorafenib, a small molecule and broad-spectrum inhibitor, is a standard systemic therapy for advanced HCC and improves a median overall survival of 6.5 months 5. Recent advances in immunotherapy and targeted therapy have substantially impacted HCC treatment 6. Notably, immune checkpoint inhibitors such as nivolumab and pembrolizumab, which target PD-1, have been approved as second-line therapies for patients pretreated with sorafenib 7. Additionally, targeted therapies including regorafenib, cabozantinib, and ramucirumab have received approval for use in similar patient populations 8. Combination strategies are also being explored to enhance therapeutic efficacy in HCC. For example, the combination of atezolizumab and bevacizumab has demonstrated superior outcomes in sorafenib-pretreated patients compared to treatment with nivolumab or pembrolizumab alone 9. Furthermore, the combination of sorafenib and doxorubicin has been shown to improve overall survival to 10.5 months 10. Despite these advancements, the efficacy of current treatments is often limited by tumor heterogeneity and individual variations, leading to the development of resistance to chemotherapy or targeted therapies and adverse reactions in a large proportion of patients 11. Additionally, the response rates to most approved drugs remain below 20%, largely due to the highly heterogeneous genetic alterations in tumors 12. Common genetic alterations in HCC include somatic mutations or copy number variations in the TERT promoter and the TP53, CTNNB1, CDKN2A, and CCND1 genes 13. Given the increasing morbidity and mortality rates of HCC, there is an urgent need for novel therapeutic approaches to improve oncological outcomes and enhance patients' quality of life.

Patchouli alcohol (PA) is a tricyclic sesquiterpene derived from *Pogostemon cablin* and is a small molecule with a molecular weight of 222.37 g/mol. It has been reported that PA improves obesity by suppressing adipogenesis and fat accumulation in adipocytes, and this could be mediated by increasing the expression and activation of β-catenin 14. Furthermore, PA ameliorated high-fat diet (HFD)-induced hepatic steatosis by attenuating endoplasmic reticulum (ER) stress and regulating low-density lipoprotein (VLDL) metabolism 15. The literature has confirmed the protective effects of PA on improving dextran sodium sulfate (DSS)-induced and 2,4,6-trinitrobenzenesulfonic acid (TNBS)-induced colitis by reducing inflammatory cytokines, maintaining intestinal integrity, and repairing macroscopic colon lesions 16, 17. PA eliminates *Helicobacter pylori* and inhibits intracellular urease activity, and this contributes to its immense potential in *H. pylori*-induced gastritis 18. It also exhibits gastroprotective effects in ethanol, indomethacin, and stress-induced ulcers 19. As an anticancer agent, PA exerts growth inhibitory effects against colon cancer by inhibiting histone deacetylase (HDAC) activity and c-myc expression to activate p21 and downregulate cyclin D1 and cdk4 20. Additionally, PA triggers apoptosis and cell cycle arrest by blocking the phosphorylation of EGFR pathways and activating the JNK and p53/p21 pathways in A549 and vincristine-resistant A549 lung cancer cells 21, 22. PA attenuates the migratory ability of B16F10 cells by upregulating E-cadherin and downregulating p-Smad2/3, vimentin, and matrix metalloproteinases (MMPs) expression 23. However, its antihepatoma properties and underlying molecular mechanisms are not fully understood. Consequently, the purpose of the present study was to elucidate the extensive antihepatoma capacities of PA and its combination treatment with sorafenib *in vitro* and *in vivo* to provide a new therapeutic agent and strategy for HCC treatment.

## Materials and Methods

### Cell culture and chemicals

HepG_2_ (human HCC), Mahlavu (human HCC), J5 (human HCC), Huh7 (human HCC), BNL CL.2 (mouse liver embryonic cell), SVEC (mouse vascular endothelial cell), and MDCK (canine epithelial kidney cell) cells were purchased from the American Type Culture Collection (Manassas, VA, USA) or the Bioresource Collection and Research Center (Hsinchu, Taiwan). The cells were cultured in DMEM or RPMI-1640 containing 10% FBS (Gibco, Mexico), 1% sodium pyruvate, 1% HEPES, and 1% penicillin/streptomycin at 37 °C in a humidified incubator with 5% CO_2_. All of the cell culture reagents were purchased from Thermo Fisher Scientific (Waltham, MA, USA). Femtopath TP53 Exon8 Primer Set (HongJing Biotech., New Taipei City, Taiwan) analysis was performed on HepG_2_ and Mahlavu cells to assess the status of TP53. Patchouli alcohol (PA purity > 98%) was purchased from ChemFaces Biochemical Co. Ltd. (Wuhan, Hubei, China). Sorafenib (SOR) was purchased from LC Laboratories (Woburn, MA, USA). PA and SOR were prepared as 225 mM and 108 mM stock solutions in DMSO and stored at -20°C. For subsequent experiments, the stock solutions were diluted in complete medium to the indicated concentrations, with the final DMSO content kept below 0.5%. The reagents 5-Flurouracie (5-FU), etoposide (VP-16), propidium iodide (PI), RNase, NAC, DCF-DA, and MTT were purchased from Sigma-Aldrich, Inc. (MO, USA). Doxorubicin was purchased from Toku-E (Bellingham, WA, USA). JC-1 and DAPI was purchased from AAT Bioquest, Inc. (Sunnyvale, CA, USA).

### Cell proliferation assay

Cell viability was assessed using the MTT-based colorimetric assay. Cells (5 ×10^3^ cells/100 μl) were seeded into 96-well plates and treated with serial concentrations of drugs for 24, 48, and 72 h. Control wells were treated with an equivalent volume of DMSO, resulting in a final concentration of 0.5% in the medium. The absorbance was determined using a SpectraMax M5 microplate reader (San Jose, CA, USA) at 550 nm, and cell viability was presented as the percentage of live cells relative to the control. All experiments were performed in triplicate and independently, and the results are expressed as means ± SD.

### Analysis of cell cycle distribution

The cell cycle was analyzed using a FACSCalibur (Franklin Lakes, NJ, USA). HepG_2_ and Mahlavu cells (2 × 10^6^) were seeded into 10 cm dishes, cultured overnight, and then treated with 89.9 and 112.4 μM PA for 0, 6, 12, 24, and 48 h, respectively. After treatment, the cells were harvested and incubated in a solution containing propidium iodide (PI, 40 μg/ml) and RNAase overnight at 4 °C and then analyzed for FL2 intensity using FACSCalibur. The proportions of cells in the G0/G1, S, and G2/M phases and the percentage of cells in the subG1 phase were evaluated using FlowJo 7.6.1 (Ashland, OR, USA).

### Western blotting

Cells (2 × 10^6^) were seeded into 10 cm dishes, incubated overnight, and treated with the indicated concentrations of PA for 0, 6, 12, 24, or 48 h. After cell collection, RIPA buffer (Bio Basic Inc., Toronto, Canada) containing a protease inhibitor (Amresco Inc., USA) and phosphatase inhibitor cocktail (Bionovas, Toronto, Canada) was used to lyse the cell pellets. Protein concentration was determined by BCA Protein Assay Reagent (Thermo Fisher Scientific, Waltham, MA USA), and 20 μg of total lysates was used for analysis of western blotting. Proteins were resolved on 8-12.5% SDS polyacrylamide gels, transferred to 0.2 μm polyvinylidene difluoride blotting membranes (Pall Corporation, Washington, NY, USA), and probed with the indicated antibodies. All antibodies were obtained from Santa Cruz Biotechnology (Dallas, TX, USA) or iReal Biotechnology Co., Ltd. (Hsinchu, Taiwan). The bands were visualized using a T-Pro LumiFast Plus Chemiluminescence Detection Kit (T-Pro Biotechnology, Taipei, Taiwan). The signals were analyzed and quantified using ImageJ software (version 1.8.0; NIH, Bethesda, MD, USA).

### Measurement of ROS production

HepG_2_ and Mahlavu cells (2 × 10^5^) were seeded into 6 well plates overnight, and then treated with 89.9 and 112.4 μM PA for 0, 1, 3, 6, 9, and 12 h, respectively. In contrast, cells were pretreated with *N*-Acetyl-L-cysteine (NAC, 12.3 mM) for 1 h and then treated with the indicated concentration of PA for 12 h. After treatment, the cells were stained with DCF-DA according to the manufacturer's instructions for flow cytometry. Flow cytometry data were analyzed using FlowJo 7.6.1. and are presented as a percentage of treatment relative to controls.

### Detection of mitochondrial membrane potential

The mitochondrial membrane potential (MMP) was measured by flow cytometry using JC-1 staining. HepG_2_ and Mahlavu cells (2 × 10^5^) were seeded into 6-well plates overnight and treated with the indicated PA doses for 0, 3, 6, 12, and 24 h. Cells were stained with JC-1 that was performed according to the manufacturer's instructions and detected and analyzed using FACSCalibur and FlowJo 7.6.1.

### TUNEL staining

A TUNEL assay kit (Terminal Transferase dUTP Nick End labelling Kit; Sigma, St. Louis, MO, USA) was used according to the manufacturer's instructions. Treated cells or tissue sections were incubated with 3% H_2_O_2_ in methanol, permeabilized with 0.1% Triton X-100 in 0.1% sodium citrate on ice, incubated with TUNEL reagent, and counterstained with PI (10 μg/ml). The slides were observed and photographed using a fluorescence microscope (ZEISS AXioskop2; Bremen, Germany) at a ×400 magnification.

### Scratch wound healing assay

HepG_2_ cells (4 × 10^5^) were seeded into 6-well plates and allowed to grow to 95% confluence. A scratch wound was generated using a P200 micropipette tip, and floating cells were removed by washing with PBS. Cells were treated with PA (0, 45, 67.5, 89.9, or 112.4 μM) and then observed and photographed after treatment for 24 and 48 h.

### Boyden chamber assay

The upper and lower chambers were separated using nitrocellulose filters (pore size, 8 μm; GVS North America, Inc.) coated with 20 μl of Matrigel (0.5 mg/ml; Corning Corp.) on the upper surface. The bottom well contained complete medium that was supplemented with 10% FBS. HepG_2_ cells (5 × 10^4^) were seeded into serum-free medium in the upper chamber and treated with PA (0, 22.5, 45, 67.5, or 89.9 μM). After incubation for 24 h, the cells on the lower surface of the filter were fixed with methanol on ice, stained with 0.1% crystal violet, and then observed and photographed using a microscope and digital microscope camera.

### Colony formation assay

HepG_2_ cells were seeded at a density of 500 cells/well into a 10 cm dish and treated with PA (0, 45, 67.5, 89.9, or 112.4 μM) for 24 h. After the removal of PA, the treated cells were incubated in complete medium for 10 days. At the end of incubation, the colonies were washed with PBS, fixed with 10% formalin, stained with 0.1% crystal violet, counted, and calculated as percentage of treatment relative to control.

### RNA extraction and Semiquantitative RT-PCR

Total RNA was extracted using the TRIzol method (iNtRON Biotechnology Co., Ltd., New Taipei City, Taiwan), and 5 μg of RNA was converted into cDNA using a HiSpec Reverse Transcriptase kit (Yeastern Biotech Co., Ltd., New Taipei City, Taiwan) following the manufacturer's instructions. A total of 40 cycles of PCR (95 °C for 30 s, 55-56 °C for 30 s and 72 °C for 30 s) were performed on a Thermo Cycler PX2 PCR instrument (Thermo Fisher Scientific, Inc.) using 5× PCR MasterMix (Raibow Biotechnology Co., Ltd., Taipei, Taiwan), cDNA template, and primers (Supplementary Table 2). PCR products were electrophoresed on 2% agarose gels, stained with ethidium bromide, photographed using an UV transilluminator (AlphaImager HP, CA, USA), and quantified for the intensity of each band using ImageJ software (version 1.8.0; NIH, Betlesda, MD, USA).

### Evaluation of combination effect *in vitro*

HepG_2_ cells (5 ×10^3^) were seeded into 96-well plates and treated with serial concentrations of PA (0-89.9 μM) and sorafenib (SOR, 0-8.6 μM) for 24 and 48 h, and this was followed by detection for cell viability using MTT assay. The effects of the drug combinations were evaluated using a combination index (CI), including synergism (CI < 1), additive effect (CI = 1), and antagonism (CI > 1), and the data were generated automatically using CompuSyn software (ComboSyn, Inc., Paramus, NJ. USA).

### Subcutaneous HCC xenograft model

Six-week-old nude BALB/c mice were obtained from the National Laboratory Animal Breeding and Research Center (Taipei, Taiwan). The animal study was performed at the Chung Shan Medical University (CSMU) and approved by the Institutional Animal Care and Use Committee of CSMU (approval no. CSMU-IACUC-2599). HepG_2_ cells (1×10^6^) in 100 μl PBS were injected subcutaneously into the right flanks of mice that were randomly divided into five groups: (1) vehicle (n=4); (2) PA (75 mg/kg, s.c.; n=4); (3) PA (150 mg/kg, s.c.; n=4); (4) sorafenib (30 mg/kg, i.p.; n=4); (5) combination treatment group (75 mg/kg PA, s.c.; 30 mg/kg sorafenib, i.p.; n=4). Five days after cell injection, mice received PA and/or sorafenib every 2 days for 3 weeks. Tumor volume and body weight were recorded, and the health status was monitored during the therapeutic procedure. When the tumor volume (L×H×W mm^3^) was > 1,500 mm^3^, the mice were sacrificed by CO_2_ asphyxiation, and tumors and organs were collected and fixed with 10% neutral formalin for analysis by immunofluorescence (IF), hematoxylin-eosin (H&E), and immunohistochemistry (IHC) staining 24.

### Computational modeling of AR and PA

The crystal structure of the human wild-type androgen receptor (PDB code: 1E3G) was obtained from the Protein Data Bank, and energies with patchouli alcohol (CID: 10955174) were calculated to predict the docking position using Auto Dock VinaPyRx-0.8 software. The 3D structural diagrams of AR and PA were visualized via EduPyMOL-v1.7.4.5 software.

### The inhibitory effects of PA on DHT-induced proliferation

HepG_2_ cells (5 × 10^3^) were cultured in 96-well plates in medium containing 2.5% FBS overnight, and this was followed by treatment with DHT (0.6 μM) and/or PA (2.8 and 5.6 μM) for 24 and 48 h. Cell viability was assessed using the MTT assay, and the results are presented as the percentage of treatment relative to the control.

### Confocal fluorescence microscopy

HepG_2_ cells were seeded onto 15 mm culture coverslips (Assistant, Germany) in 3.5 cm dishes and treated with DHT (0.6 μM) and/or PA (5.6 μM) for 12, 24, and 48 h. The treated cells were fixed with 10% neutral formalin, permeabilized with 1% NP-40 in PBS, blocked with 5% BSA, and incubated with AR primary antibody (1:200 dilution; Santa Cruz) overnight at 4°C. After washing, the cells were incubated with a secondary antibody conjugated to biotin (Santa Cruz Biotechnology) and Alexa Fluor 488-conjugated streptavidin (Jackson ImmunoResearch Inc., USA) and then counterstained with DAPI. The localization of AR in the nucleus was observed using a Zeiss LSM 510 META laser-scanning confocal microscope (Bremen, Germany).

### Statistical analysis

All values are expressed as the mean ± SD (*in vitro*) or SEM (*in vivo*). Unpaired Student's t-test and one-way ANOVA were used to identify significant differences between the control and treatment groups, and the Kaplan-Meier method was used to perform survival rate analysis. *P* < 0.05 was considered to indicate a statistically significant difference.

## Results

### PA inhibited cell growth via the induction of cell cycle arrest in HCC cells

**Figure 1:**
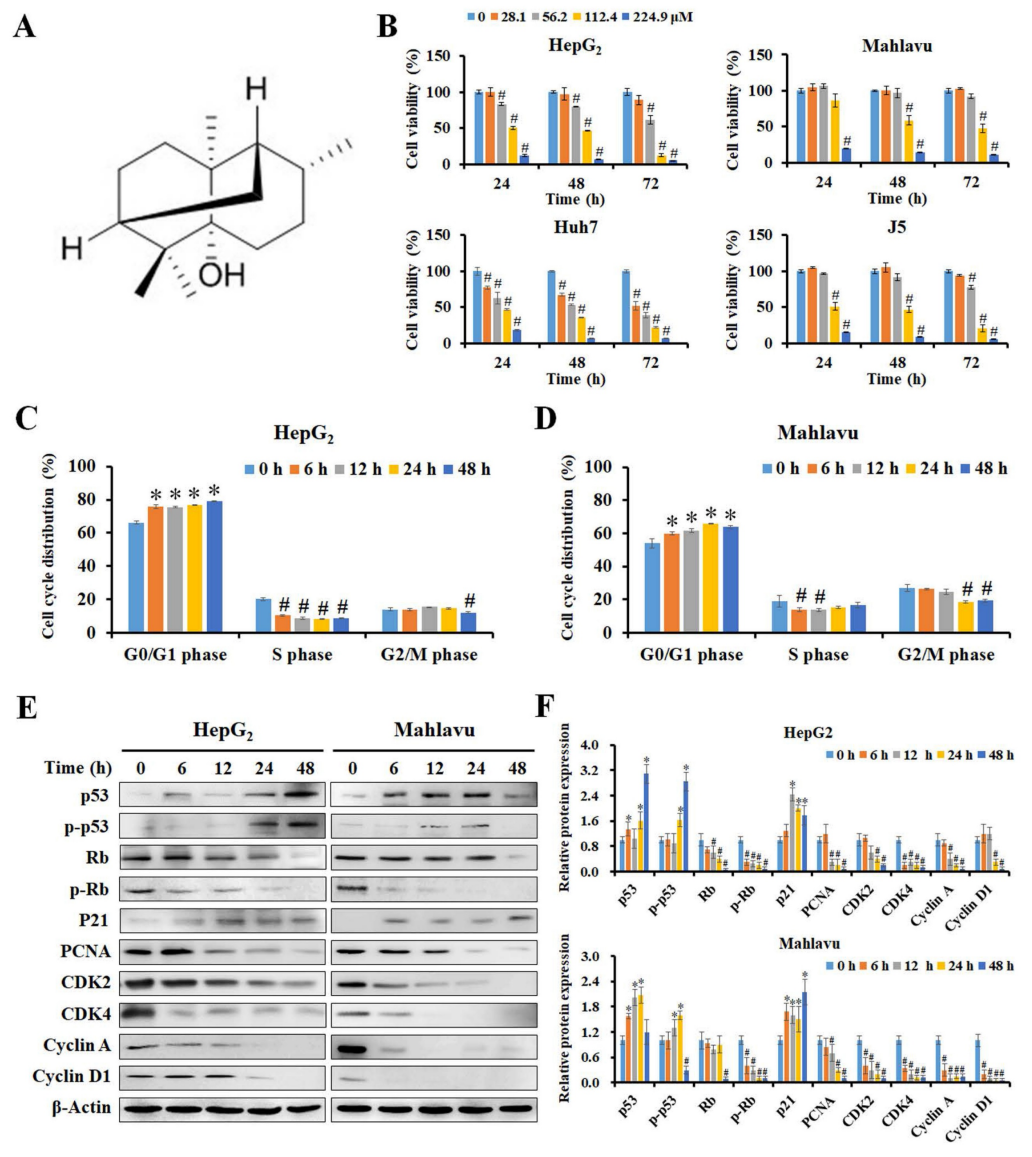
** PA inhibited cell growth by inducing cell cycle arrest in HCC cells.** (A) The chemical structure of PA from the database of ChemFaces. (B) Cells were treated with PA (0-224.9 μM) for 24, 48, and 72 h, and cell viability was assessed using MTT-based colorimetric assays. (C and D) HepG_2_ and Mahlavu cells were treated with 89.9 and 112.4 μM PA for 0-48 h, respectively. The cell cycle distribution was detected and evaluated using flow cytometry and FlowJo 7.6.1. software. (E and F) The PA-treated cells were collected and lysed, and the total proteins were analyzed for the expression of cell cycle-associated proteins by western blotting. The blots were quantified using ImageJ software, and data are expressed as mean ± SD of at least three independent experiments. *P < 0.05, versus control with significant increase. ^#^*P* < 0.05, versus control with significant decrease. Rb, retinoblastoma protein. PCNA, proliferating cell nuclear antigen. CDK, cyclin-dependent kinase.

The chemical structure of PA with a molecular weight of 222.37 g/mol is presented in Figure 1A, and its purity was greater than 98%. We first examined the inhibitory effect of PA on HCC cells using the MTT assay, and the results revealed that PA reduced the viability of HCC cells in a time- and dose-dependent manner (Figure 1B), while this effect was not evident in normal cells (Supplementary Figure 1A). Table 1 indicates that the 50% inhibitory concentration of PA ranged from 66.5 ± 3.8 μM to 146 ± 10.6 μM in HCC cells and 262.8 ± 0.2 μM to 328.6 ± 1.3 μM in normal cells, respectively. These results demonstrate that PA effectively inhibited HCC cell growth and possessed lower IC_50_ values in HCC cells than those in normal cells. To further evaluate the selectivity of PA and the clinical drugs against HCC and normal cells, the selectivity index (SI) was calculated, and a value greater than one indicated good selectivity for HCC cells. The minimal SI values of PA (1.8 to 4.9) were higher than those of sorafenib (1.3 to 12), VP-16 (0.2 to 2.6), doxorubicin (0.8 to 3.3), and 5-FU (0.1 to >4.5) as presented in Supplementary Table 1, thus suggesting that PA exerted lower cytotoxicity to normal cells than did other clinical drugs during treatment.

To study the mechanisms of PA growth inhibition, the cell cycle distribution of PA-treated cells was analyzed by measuring FL2 intensity using flow cytometry (Supplementary Figure 1B and C). As presented in Figure 1C and D, PA significantly stimulated the cell population that accumulated at the G_0_/G_1_ phase and also reduced that in the S phase and G_2_/M phase in HepG_2_ and Mahlavu cells. Additionally, western blot analysis revealed that PA constantly increased the levels of p53 and phosphorylated p53 (Ser392) to activate the expression of p21 within 24 h and reduced the expression of total Rb, phosphorylated Rb (Ser24/Thr252), PCNA, CDK2, CDK4, cyclin A, and cyclin D1 (Figure 1E and F). These data indicate that PA stimulates the p53/p21 pathway and regulates Rb/p-Rb signaling to affect cell cycle distribution, thus resulting in growth inhibition of HepG_2_ and Mahlavu cells.

### PA stimulated ROS generation and contributed to apoptosis in HCC cells

**Figure 2:**
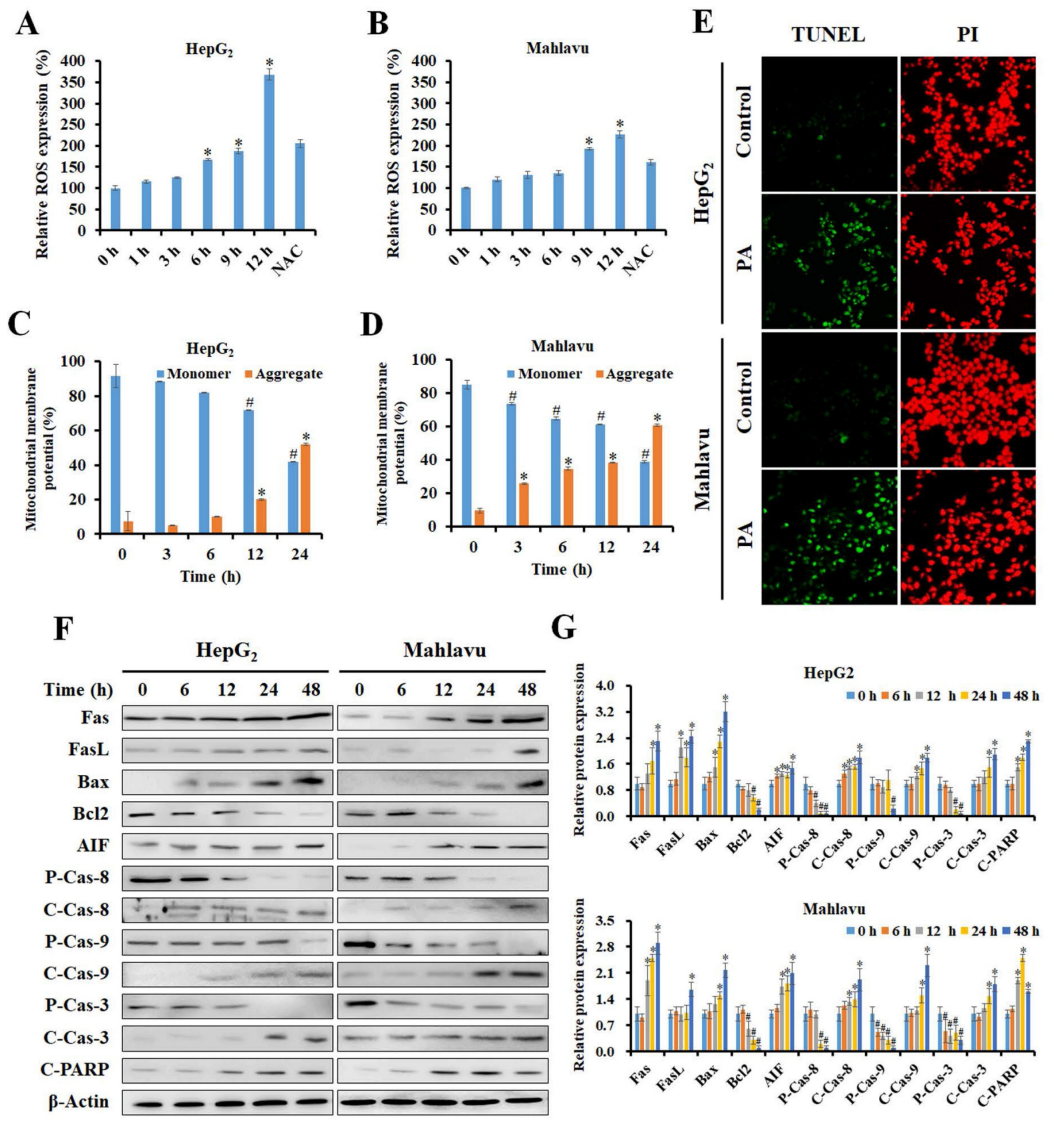
** PA stimulated ROS generation and MMP imbalance, ultimately resulting in apoptosis.** (A and B) HepG_2_ and Mahlavu cells were treated with 89.9 and 112.4 μM PA for 0-12 h, respectively, and stained with DCF-DA, and this was followed by detection of ROS levels by flow cytometry. (C and D) Cells were treated with the indicated concentrations of PA for 0-24 h and stained with JC-1 reagent to detect MMP. (E) After PA treatment for 24 h, cells were observed for apoptosis using a TUNEL assay kit and PI as a counterstain according to the manufacturer's instructions. (F and G) Total proteins from PA-treated cells were analyzed for the expression of cell apoptosis-related proteins by western blotting. All data are presented as means ± SD from three independent experiments. *P < 0.05, versus control with significant increase. ^#^*P* < 0.05, versus control with significant decrease. NAC, N-acetylcysteine. FASL, Fas ligand. AIF, apoptosis-inducing factor. P-Cas, pro-caspase. C-Cas, cleaved-caspase.

Reactive oxygen species (ROS) have been reported to trigger cell cycle arrest and apoptosis by induction of DNA damage and imbalance of mitochondria membrane potential (MMP). Previous studies have demonstrated that PA suppresses cell cycle progression by regulating the cell cycle. Next, to investigate if PA induces ROS production, cells were treated with PA, and intracellular ROS levels were detected using DCF-DA staining and flow cytometry. The results demonstrated that PA significantly stimulated ROS production within 12 h, and this was blocked by N-acetylcysteine (NAC) treatment (Figure 2A and B). JC-1 staining and flow cytometric analysis of HepG_2_ and Mahlavu cells after PA treatment revealed increased aggregate levels accompanied by decreased monomer levels, thus indicating that PA caused an imbalance in MMP (Figure 2C and D).

To determine the extent of PA-induced apoptosis, the TUNEL assay was performed. The results revealed that treatment with PA for 24 h effectively induced apoptosis in both HepG_2_ and Mahlavu cells (Figure 2E). Next, we examined the expression of key regulatory proteins that control apoptosis initiation and their effects in response to PA treatment by western blotting. PA activated the extrinsic apoptotic pathway by mediating an increase in the expression of Fas and FasL proteins and cleavage of caspase-8 in both HCC cell lines (Figure 2F and G). In contrast, PA rapidly increased the ratio of Bax and Bcl2 proteins and the expression of cleaved-caspase-9 protein, thus resulting in the activation of the intrinsic apoptotic pathway. Ultimately, caspase-3 is activated and triggers the cleavage of poly(ADP-ribose) polymerase (PARP) that is considered a hallmark of apoptosis. The expression of apoptosis-inducing factor (AIF), a caspase-independent death effector that triggers chromatin condensation and DNA fragmentation, is enhanced after PA treatment. Consequently, PA stimulated ROS production, induced MMP imbalance, and activated caspase-dependent and-independent apoptotic pathways in HepG_2_ and Mahlavu cells.

### Effects of PA on migration, invasion, and clonogenic ability of HCC cells

**Figure 3:**
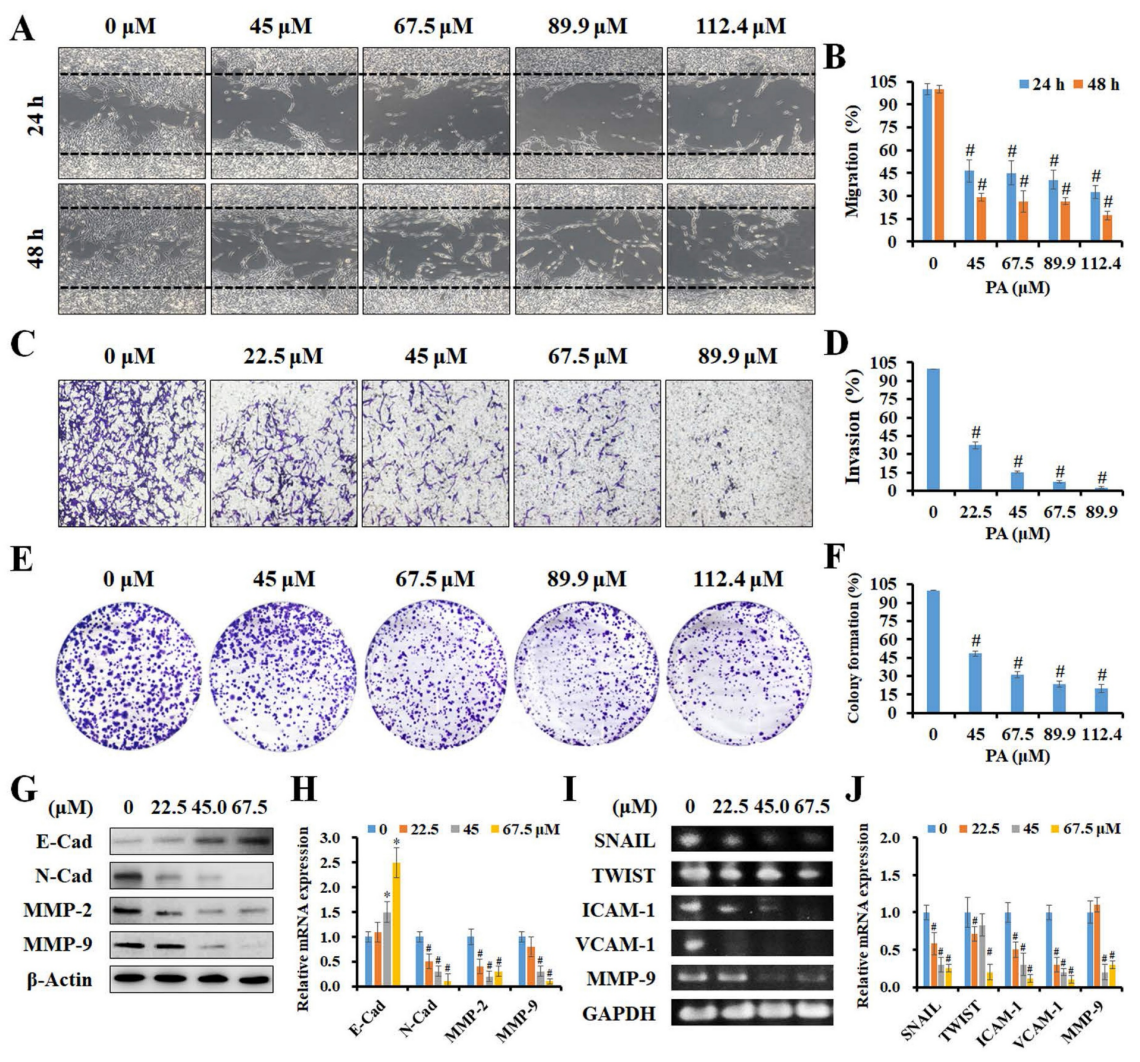
** PA attenuated the capability of migration and invasion in HCC cells.** (A and B) A scratch wound healing assay was performed on HepG_2_ cells treated with PA (0-112.4 μM) for 24 and 48 h. (C and D) Boyden chamber assay of HepG_2_ cells after PA treatment (0-89.9 μM) for 24 h, after which the invasive cells were stained, photographed, and quantified. (E and F) Cells were treated with PA (0-112.4 μM) for 24 h and then cultured in drug-free medium for another 10 days. The colonies were fixed, stained, and counted (>100 cells). (G-J) The cells were treated with PA (0-67.5 μM) for 24 h and analyzed for the expression of metastasis-related proteins and mRNA using western blotting and semi-quantitative RT-PCR, respectively. Data are presented as means ± SD in three technical replicates. *P < 0.05, versus control with significant increase. ^#^*P* < 0.05, versus control with significant decrease. Cad, cadherin. MMP, matrix metalloproteinase. ICAM-1, intercellular adhesion molecule 1. VCAM-1, vascular cell adhesion molecule 1.

To further investigate if PA inhibited the metastatic ability of HCC cells, *in vitro* scratch wound healing, invasion, and colony formation assays were performed. As presented in Figure 3A and B, higher concentrations of PA resulted in a greater number of migrated cells (%), thus indicating that PA treatment dose-dependently inhibits the migration of HCC cells. Invasion capacity was also evaluated in control cells and in cells treated with PA. As presented in Figure 3C, a significant decrease in the percentage of invasive cells (%) was observed following PA treatment. The quantification of results revealed that the invasiveness of HCC cells decreased to 37.2%, 15.3%, 7.4%, and 2.6%, respectively, after PA treatment (22.5-89.9 μM) for 24 h (Figure 3D). Next, we examined the ability of a single adherent cell to expand into a clonal population, and the results demonstrated that the number of colonies was markedly reduced to 48.5%, 31.0%, 23.6%, and 20.1% in cells treated with PA (45-112.4 μM) compared to that of the control (Figure 3E and F). Finally, we investigated the underlying mechanisms of PA in HCC metastasis, including epithelial-mesenchymal transition (EMT)-related proteins and matrix metalloproteinases (MMPs). The results demonstrated that the expression level of E-cadherin increased and those of N-cadherin, MMP-2, and MMP-9 decreased with increasing PA concentrations (Figure 3G and H) as detected by western blotting. Additionally, semi-quantitative RT-PCR indicated that the mRNA expression levels of SNAIL, TWIST, ICAM-1, VCAM-1, and MMP-9 were downregulated by PA treatment (Figure 3I and J). Taken together, these results demonstrate that PA suppresses HCC cell metastasis by inhibiting EMT and MMPs.

### PA combined with sorafenib exhibited synergistic effects against HCC cells

**Figure 4:**
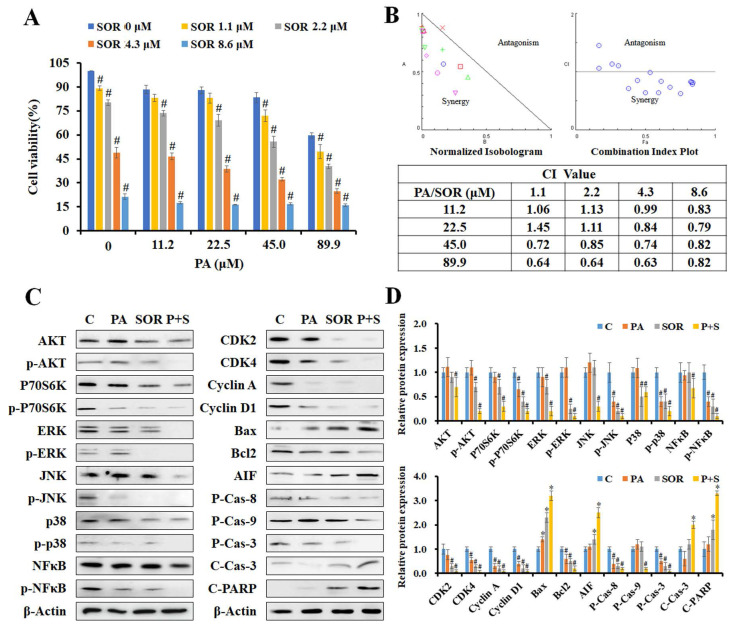
** PA combined with sorafenib exerted synergistic effects in HepG_2_ cells.** (A) The cell viability of HepG_2_ cells after treatment with PA (0-89.9 μM) and/or sorafenib (0-8.6 μM) were determined using MTT assay. (B) Normalized isobologram, combination index plot, and combination index (CI) of PA combined with sorafenib were generated using CompuSyn software. (C and D) HepG_2_ cells were treated with PA (89.9 μM), sorafenib (4.3 μM), or drug combination for 24 h. After treatment, the cells were harvested and prepared for western blot analysis for AKT and MAPK signaling and cell cycle and apoptosis-associated proteins. Data are presented as the mean ± SD. from three independent experiments. *P < 0.05, versus control with significant increase. ^#^*P* < 0.05, versus control with significant decrease. P-Cas, pro-caspase. C-Cas, cleaved-caspase.

To determine the effects of PA combined with sorafenib, HepG_2_ cells were treated with serial concentrations of PA (11.2-89.9 μM) and sorafenib (1.1-8.6 μM) for 24 h, and we detected cell viability using the MTT assay. The combination of PA and sorafenib reduced cell viability compared to that of PA or sorafenib alone (Figure 4A). To further evaluate the effects of the drug combination, the normalized isobologram and combination index (CI) were analyzed using CompuSyn software. As indicated by each point of the normalized isobologram or CI plot for the combination of PA and sorafenib, most points fell within the synergy region, thus indicating that the combined use of the two drugs exerted synergy in HepG_2_ cells (Figure 4B). The CI values of combination treatment at higher concentrations (PA >45 μM or sorafenib >4.3 μM) were less than 1, particularly for 89.9 μM PA combined with 4.3 μM sorafenib with an IC value of 0.63.

Subsequently, we further examined the effects of PA on AKT, MAPK, cell cycle, and apoptosis associated protein expression in cells treated with 89.9 μM PA and/or 4.3 μM sorafenib for 24 h by western blotting (Figure 4C and D). The results revealed that PA combined with sorafenib effectively decreased the levels of AKT, P70S6K, ERK, JNK, p38, NFκB, and their phosphorylated proteins, thus indicating that the combination treatment strengthened the suppression of AKT, MAPK and NFκB signaling to reduce cell proliferation, growth, and survival in HepG_2_ cells. Moreover, the expression of Rb and p-Rb proteins and their downstream regulators, including CDK2, CDK4, cyclin A, and cyclin D1, was significantly reduced after combination treatment. Additionally, combination treatment significantly enhanced the ratio of Bax to Bcl2, the cleavage of pro-caspase-8, pro-caspase-9, pro-caspase-3, and PARP, and the expression of AIF compared to that induced by a single drug. These results suggest that PA and sorafenib exerted synergistic effects on growth inhibition and the regulation of growth, cell cycle, and apoptosis-related proteins.

### PA exerted significant antitumor activity against HCC in vivo

**Figure 5:**
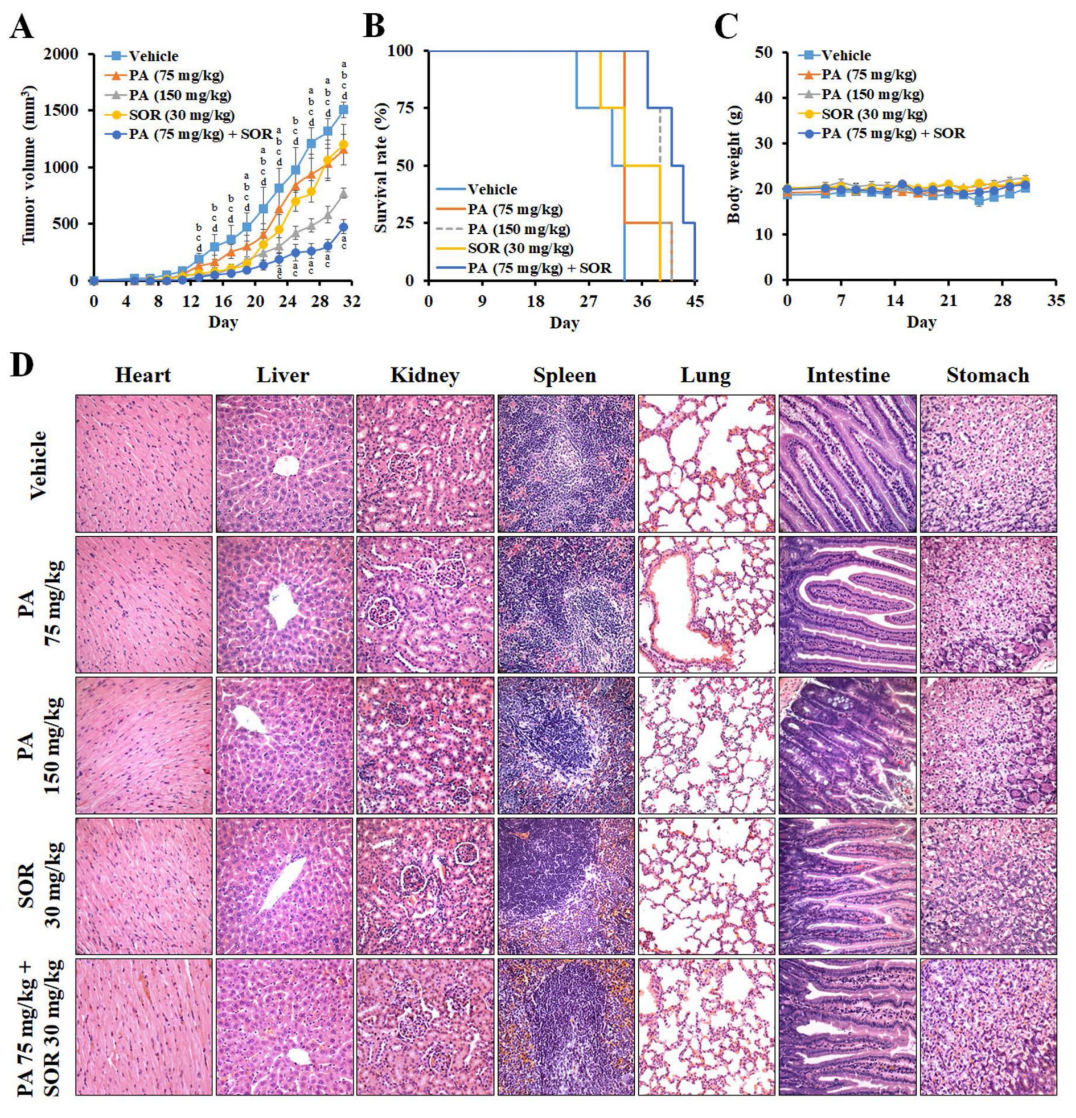
** PA suppressed tumor growth in a human HCC xenograft animal model.** (A-C) Mice bearing HepG_2_ HCC cells were treated with vehicle, 75 mg/kg PA (s.c.), 150 mg/kg PA (s.c.), 30 mg/kg sorafenib (i.p.), and drug combination (75 mg/kg PA combined with 30 mg/kg sorafenib) once every 2 days for 3 weeks. Tumor volume and body weight were measured once every 2 days. When the tumor size was greater than 1,500 mm^3^, the mice were sacrificed, and the organs and tumor were collected for histological analysis. The statistical analysis of survival rate was performed using the Kaplan-Meier method (*P* < 0.05). Data are presented as mean ± SEM. ^a^*P* < 0.05, versus 75 mg/kg PA. ^b^*P* < 0.05, versus 150 mg/kg PA. ^c^*P* < 0.05, versus sorafenib. ^d^*P* < 0.05, versus drug combination. (D) No significant differences were observed between the groups in terms of histological morphology as analyzed by hematoxylin and eosin (H&E) staining. The experiments were repeated two times. SOR, sorafenib.

To further evaluate the antitumor effect of PA *in vivo*, we treated HepG_2_ tumor-bearing mice with PA once every two days at doses of 75 or 150 mg/kg. In comparison to vehicle, the tumor volumes were reduced from 1,505 ± 72 to 1,155 ± 134, 771 ± 45, 1,200 ± 177, and 476 ± 65 mm^3^ at day 31, and lifespan was prolonged from 33 to 41, 41, 39, and 45 days after treatment with 75 mg/kg PA, 150 mg/kg, 30 mg/kg sorafenib, and the drug combination (75 mg/kg PA + 30 mg/kg sorafenib), respectively (Figure 5A and B). Therefore, PA inhibits tumor growth and acts synergistically with sorafenib to diminish the proliferation of HCC cells. PA alone or in combination also extended lifespan, thus supporting survival benefits in the HCC xenograft model.

To estimate the systemic toxicity of PA *in vivo*, the body weights of the mice were monitored, and no significant alterations were observed between the groups (Figure 5C). Histological examination of hematoxylin and eosin (H&E)-stained sections revealed no obvious irreversible morphological changes in the heart, liver, kidney, spleen, lung, intestine, or stomach (Figure 5D). Furthermore, we assessed the changes in blood cells, including white blood cells (WBC), red blood cells (RBC), platelets (PLT), hemoglobin (HGB), and hematocrit (HCT), and these values were within the normal ranges (Supplementary Figure S1). Blood urea nitrogen (BUN), creatinine, total bilirubin, aspartate aminotransferase (AST), and alanine transaminase (ALT) were within normal ranges. These results demonstrated that PA doses combined with sorafenib were well tolerated *in vivo*.

**Figure 6:**
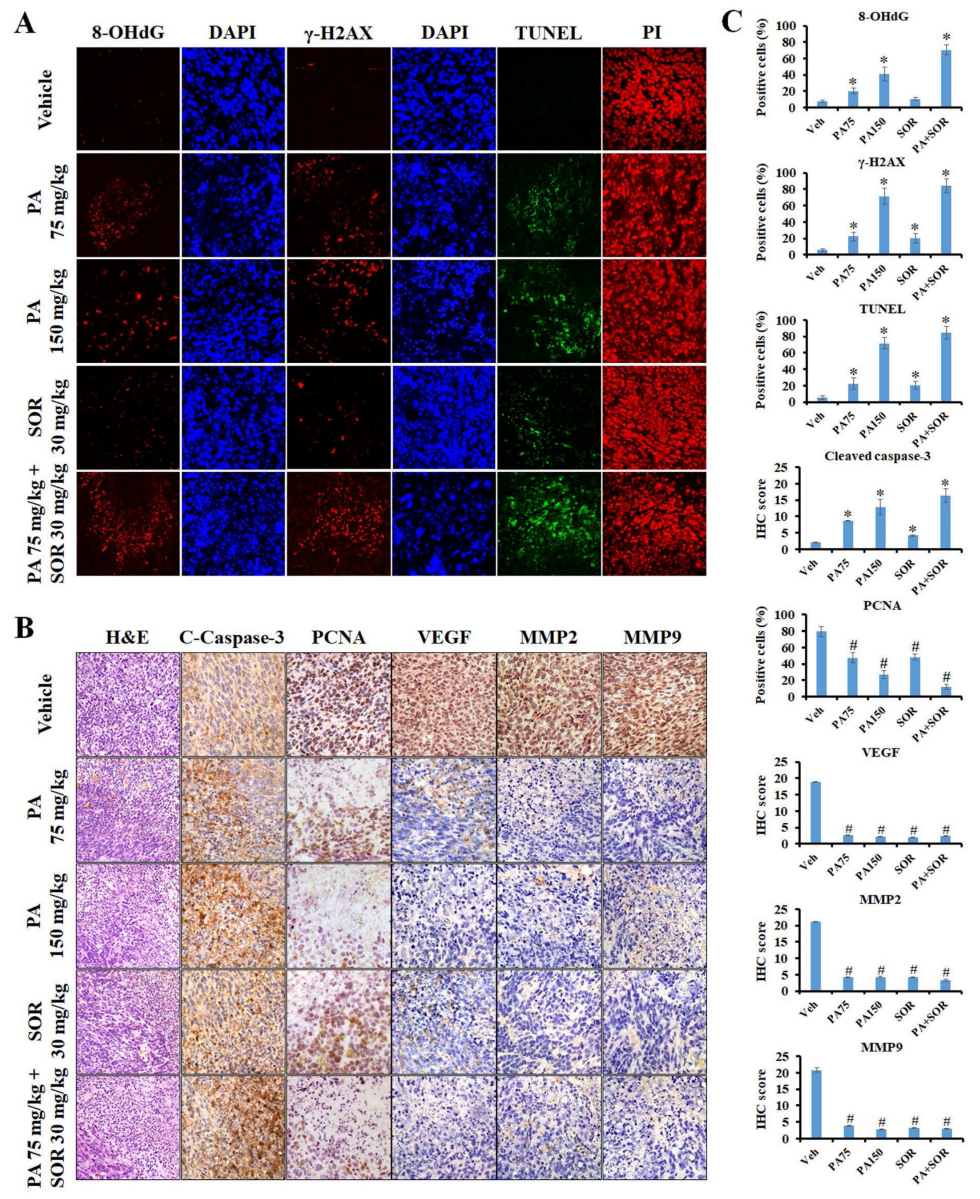
** PA inhibited tumor progression and triggered apoptosis through the induction of ROS production and DNA damage.** (A and B) The anticancer mechanism of PA *in vivo* was determined using immunofluorescence, tissue TUNEL, H&E, and immunohistochemistry staining. DAPI, PI, and hematoxylin stains were used as counterstains, respectively. (C) Protein expression in tumor tissues was quantified and presented as IHC scores or percentages, respectively. All data are reported as means ± SEM. *P < 0.05, versus control with significant increase. ^#^*P* < 0.05, versus control with significant decrease. 8-OHdG, 8-hydroxy-2-deoxyguanosine. PCNA, proliferating cell nuclear antigen.

An immunofluorescence assay was performed in tumor tissues to further investigate if PA exerts its anticancer effects by triggering ROS generation and DNA damage *in vivo*. The results revealed that the protein expression levels of the DNA oxidative stress maker 8-OHdG and the DNA damage marker γ-H2AX increased sequentially in the tumor tissues of the 75 mg/kg PA, 150 mg/kg PA, and PA in combination with sorafenib treatment groups compared to those of the vehicle treatment group (Figure 6A and C). Furthermore, TUNEL-positive cells and cleaved caspase-3 protein expression were conspicuously exaggerated in a dose-dependent manner after PA treatment, and drug combination administration enhanced these effects compared to those observed in response to PA or sorafenib alone. These results demonstrated that PA alone or in combination with sorafenib facilitated ROS production, thus resulting in DNA damage and induced cell apoptosis, and this was consistent with the *in vitro* results (Figure 6A-C). Immunohistochemical staining was performed to determine the molecular mechanisms underlying the regulation of tumor growth and metastasis. Compared to the untreated counterpart (vehicle group), PA treatment led to a substantial decrease in the expression levels of proliferating cell nuclear antigen (PCNA), vascular endothelial growth factor (VEGF), MMP-2, and MMP-9 (Figure 6B and C), and this was strengthened by combined with sorafenib. Overall, the findings clearly indicated that PA exerted strong antitumor activity via the induction of ROS-mediated apoptosis and also caused the inhibition of tumor growth and metastasis with low toxicity *in vivo*.

### PA suppressed cell growth via specifically targeting androgen receptors in HCC cells

**Figure 7:**
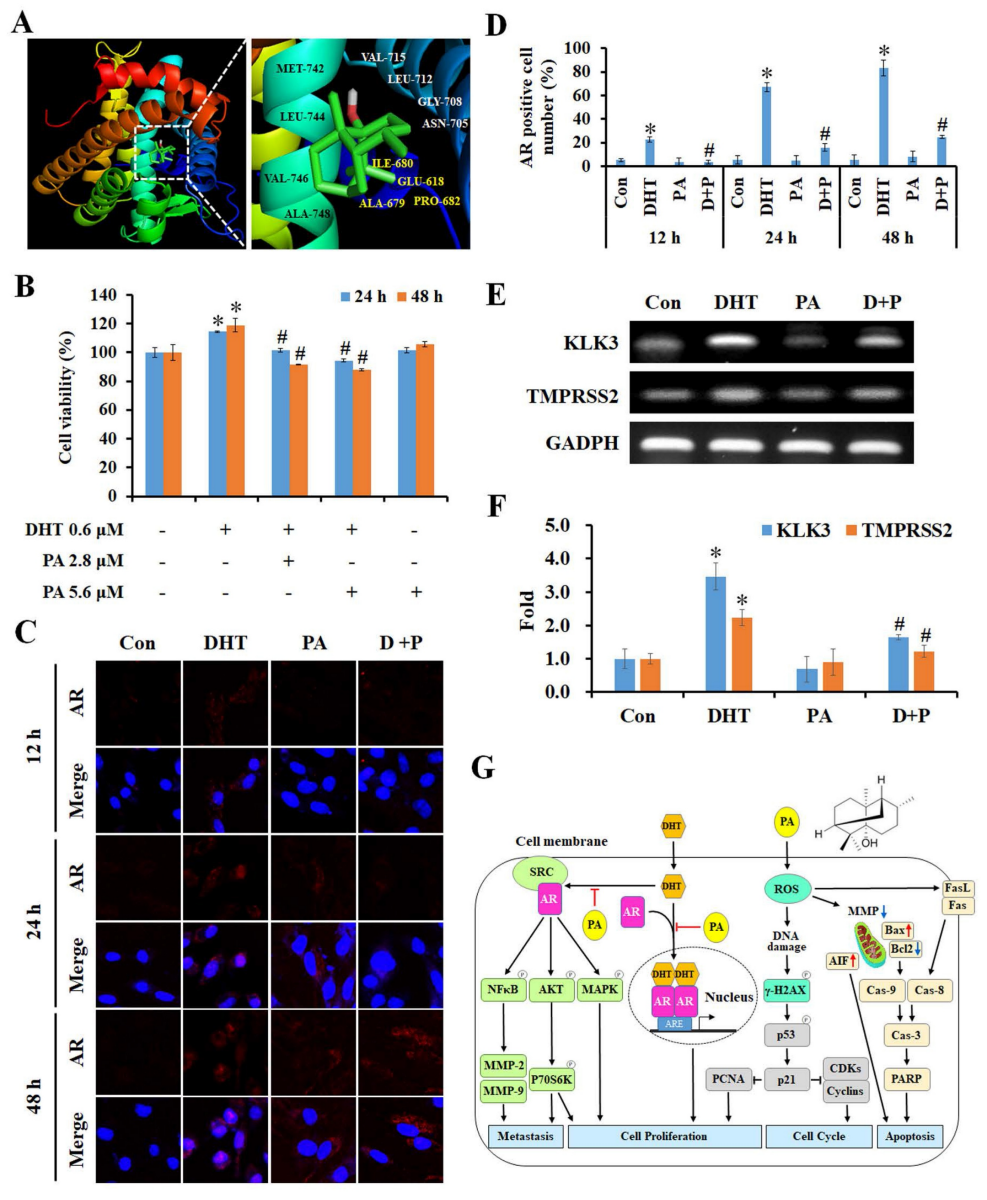
** Specific binding of PA to AR inhibited DHT-induced proliferation by blocking downstream gene expression.** (A) Putative binding modes of PA and AR were predicted through molecular modeling. (B) The cell viability of HepG_2_ cells after DHT and/or PA treatment was detected by MTT assay. (C and D) The AR translocation of cells treated with DHT (0.6 μM) and/or PA (5.6 μM) for 0-48 h was observed and quantified by confocal microscopy. (E and F) The expression of AR downstream genes was measured using semi-quantitative RT-PCR. (G) A schematic diagram describing the potential anticancer mechanisms of PA against HCC. Data are presented as means ± SD from three independent experiments. *P < 0.05, versus control with significant increase. ^#^*P* < 0.05, versus DHT with significant decrease. D+P, DHT+PA.

Next, we used the SwissTargetPrediction and SwissDock databases to identify and analyze the predicted potential target protein of PA, and this was the androgen receptor (AR). The crystal structure of human AR (PDB ID: 1E3G) was used to predict the position of the PA docking using Auto Dock VinaPyRx-0.8 software, and the computational modeling structure of AR and PA was visualized using EduPyMOL-v1.7.4.5 software (Figure 7A). To examine the competitiveness of AR between PA and dihydrotestosterone (DHT), cells were treated with DHT and/or PA, and cell viability was assessed using the MTT assay. The results revealed that DHT increased the proliferation of HCC cells; however, this effect was blocked by PA in a dose-dependent manner (Figure 7B). Moreover, PA inhibited DHT-induced AR translocation from the cytoplasm to the nucleus (Figure 7C and D) and downregulated the mRNA expression of the downstream genes kallikrein-related peptidase 3 (KLK3) and transmembrane protease serine 2 (TMPRSS2) (Figure 7E and F). Taken together, one of the anti-HCC mechanisms of PA may be binding to AR and obstructing its nuclear translocation, thus leading to decreased expression of downstream genes.

## Discussion

Patchouli alcohol (PA) accounts for approximately 30% of steam distillation-extraction from *Pogostemon cablin* 25. It has been determined to play a role in several bio-functions in gastrointestinal disease; however, its anticancer activity has only been investigated in colon cancer, lung cancer, and melanoma 20-23. Additionally, its toxicity during therapy, effects of combination with clinical drugs, and molecular targets have not fully examined. In the present study, PA was determined to inhibit HCC growth and exert a better selective index than that of chemotherapeutic drugs (VP-16, doxorubicin, and 5-FU) in normal cells. These results suggest that PA can attack tumor cells and is less cytotoxic to normal cells, thereby reducing the risk of potential side effects during therapy. Subsequently, PA was observed to efficiently suppress HCC cells by inducing cell cycle arrest in the G_0_/G_1_ phase, and this was accompanied by a decline in S and G_2_/M phases. HepG_2_ cells expressing WT p53 continuously activated p53 and significantly increased p21 expression following PA treatment. Mahlavu cells with mutant p53 exhibited increased p53 and p-p53 protein expression within 24 h and decreased expression at 48 h. However, p21 levels persisted for 48 h. Aberrations in the TP53 gene commonly occur during hepatocarcinogenesis 26. These results revealed that PA may activate p53-dependent and -independent pathways to activate p21 and block cell cycle progression in HepG_2_ and Mahlavu cells. After the activation of p53, the levels of downstream proteins, including PCNA, CDKs (CDK2 and CDK4), and cyclins (cyclin A and D1), were markedly diminished from 6 to 48 h in both HCC cell lines. Rb is a tumor suppressor that blocks the transition from G1 to S phase 27. Rb binds to E2F factors to repress their transactivation; however, when phosphorylated, E2F factors are released and the cell cycle proceeds. The results demonstrated that PA significantly repressed Rb and p-Rb levels to block the cell cycle at the G_0_/G_1_ phase and that the inhibitory trend was similar in HepG2 and Mahlavu cells. These findings provide a sufficient basis to assess the inhibitory activity of PA on cell proliferation and reveal that PA triggers p53 activation, increases p21 expression, and reduces Rb/p-Rb and CDKs/cyclins expression to interrupt cell progression at the G_0_/G_1_ phase.

Reactive oxygen species (ROS) have been reported to trigger cell cycle arrest and apoptosis by inducing DNA damage and mitochondrial membrane potential (MMP) imbalance 28. In present study, PA not only stimulated ROS production to imbalance MMP resulting in apoptosis *in vitro* but also increased the levels of the DNA oxidative stress maker 8-OHdG and the DNA damage marker γ-H2AX *in vivo*. This suggests that PA promotes ROS-induced DNA damage and MMP imbalance, ultimately leading to the induction of apoptosis. A previous study indicated that PA induces apoptosis through both the extrinsic and intrinsic apoptotic pathways in lung cancer and melanoma by regulating the caspase cascade 22, 23. It is well established that ROS upregulate FasL expression and mediate MMP imbalance to induce apoptosis activation 28. In our study, PA increased the expression of Fas and FasL, thus contributing to the cleavage of pro-caspase-8 and leading to the activation of the extrinsic apoptotic pathway in both HCC cell lines. In contrast, the ratios of Bax/Bcl2 and cleaved-caspase-9/pro-caspase-9 were enhanced, ultimately resulting in the activation of the intrinsic apoptosis pathway after PA treatment. Finally, the expression of cleaved caspase-3 was increased both *in vitro* and *in vivo*. Furthermore, during apoptosis and upon dissipation of MMP, AIF undergoes proteolysis and translocates from the mitochondria to the nucleus where it triggers partial chromatin condensation and large-scale (~ 50 kbp) DNA degradation in a caspase-independent manner 29. AIF levels are enhanced by PA treatment. Our findings revealed that PA facilitated ROS production to induce apoptosis by promoting MMP imbalance and the interaction of FasL/Fas that contributed to the activation of intrinsic and extrinsic caspase cascades, respectively, as well as accumulated levels of AIF to activate the caspase-independent pathway.

Notably, liver dysfunction and patient performance status are important parameters for evaluating therapeutic efficacy in HCC. To evaluate the safety and tolerability of PA *in vivo*, body weight assessment and hematoxylin and eosin (H&E) staining of organs in mice and blood cells and serum biochemistry analysis in rats were performed. In present study, there was no significant difference between the 75 mg/kg PA, 150 mg/kg PA, 30 mg/kg sorafenib, and drug combination (75 mg/kg PA + 30 mg/kg sorafenib) groups in terms of body weight and the histological morphology of the heart, liver, kidney, spleen, lung, intestine, and stomach after 3 weeks of treatment. Furthermore, blood cell counts (WBC, RBC, PLT, HGB, and HCT) and serum biochemistry (BUN, creatinine, total bilirubin, AST, and ALT) were within normal ranges after one dose of 150 mg/kg PA treatment for 0-24 h. Clinical features of HCC include severe loss of liver function, and this restricts therapeutic options. We demonstrated that PA administration did not damage or affect renal and liver function by examining the correlated physiological values and observing the pathological morphology of the main organs. Previous studies have demonstrated that PA prevents non-alcoholic fatty liver disease (NAFLD), ameliorates colitis, and protects against gastric ulcers 30. Importantly, PA attenuates intestinal mucositis induced by the clinical drug 5-fluorouracil, thus improving the side effects of anticancer drugs 31. Taken together, our results indicated that PA exhibited good antihepatoma activity with well-tolerated treatment and may not cause potential side effects in future clinical applications.

In humans, androgens are important sex steroid hormones involved in various cellular processes such as cell growth, metabolism, differentiation, and sexual development. Androgens, including testosterone and its metabolite 5α-dihydrotestosterone (DHT), act through the androgen receptor (AR), a 110 kDa ligand-inducible nuclear receptor with four functional domains that include a NH2-terminal transactivation domain, a central DNA-binding domain, a hinge region, and a C-terminal ligand-binding domain 32. After binding to its ligands, intracellular AR forms a homodimer and is recruited to the androgen response elements (AREs) that are present in regulatory elements on the AR-responsive target genes 33. Several studies have demonstrated that AR overexpression is significantly associated with advanced HCC, poor survival rates, and marked alterations in AR-dependent signaling, which stimulate oncogenic growth and therapeutic responses 34, 35. AR expression positively correlates with prognosis due to its metastatic and drug-resistant characteristics, highlighting AR as an emerging therapeutic target for HCC 36-38. In a ligand-independent manner, androgen-triggered activation of the AR-Src complex enhances crosstalk between multiple intracellular signaling cascades, such as the AKT and MAPK pathways, in cancers 39, 40. A previous study indicated that co-targeting the AR and AKT/mTOR pathways improves the efficacy of anti-AR therapies in HCC 41. Additionally, during tumorigenesis, ERK, p38, and JNK of the MAPK family play essential roles in regulating tumor growth and are influenced by androgen-mediated AR signaling 42. Our findings suggest that PA inhibits the expression and phosphorylation of AKT/P70S6K and MAPK, thereby blocking cell growth, survival, and migration. This supports the potential of co-targeting AR and AKT or MAPK signaling as a therapeutic strategy for HCC treatment. Furthermore, AR acts as a transcriptional coactivator for several genes, including CCND1, CCRK, CCNE, CCNE kinase, TCF7, TLR4, NFκB, SRIB, CD1, and PEG10, thereby promoting the growth of HCC (54). AR also triggers the proliferation by mediating PCNA, CDK2, Cyclin D1, miR-216a, and ADAR1 genes, attenuates the apoptosis by regulating the GRP78/BIP genes as well as maintains the property of cancer stemness by targeting EZH2, Nanog and Wnt/β-catenin genes 43. Moreover, AR stimulates migration and invasion by regulating the levels of ID1, TLR4, and Rac1, but inhibits migration and invasion by targeting Angpt2, MMP9, Circ-LNPEP, miR-325, miR-122-5p, and miR-7-5p, indicating that AR plays a dual role in HCC metastasis 43. AR signaling is implicated in the initiation of HCC associated with hepatitis B virus (HBV) infection 44. In this investigation, we observed that PA at lower concentrations exerts anti-AR activity in vitro, with significant effects observed at approximately 2.8 and 5.6 μM. Notably, PA competes with DHT to inhibit AR-induced proliferation, impede the translocation of AR into the nucleus, and suppress the expression of downstream AR genes. These findings suggest that PA presents a novel mechanism of action in suppressing HCC growth by interfering with AR signaling. Furthermore, our study revealed that PA treatment modulates the expression of proteins associated with key cellular processes, including the cell cycle (CDKs and cyclins), apoptosis, metastasis (MMPs), and growth (NFκB), underscoring its potential as an anticancer agent through the inhibition of AR signaling. Nevertheless, additional investigations are warranted to comprehensively elucidate the molecular pathways involved. Our results indicate that PA functions as a promising AR inhibitor in the context of HCC, offering potential therapeutic avenues for further exploration and development.

In conclusion, our findings indicate that PA blocks DHT-induced AR activation and nucleus translocation to downregulate the AKT, MAPK, and NFκB signaling, thus leading to a reduction in proliferation and metastasis capacity in HCC (Figure 7G). Furthermore, PA triggered ROS generation, MMP imbalance, and DNA damage, ultimately resulting in a significant accumulation of cells arrested at the G_0_/G_1_ phase via the regulation of p53/p21 and CDKs/cyclins. Simultaneously, PA induces apoptosis by activating the extrinsic (FasL/Fas/caspase-8), intrinsic (Bax/Bcl2/caspase-9), and caspase-independent (AIF) pathways. Importantly, the combination of PA and sorafenib demonstrated therapeutic efficacy against HCC *in vitro* and *in vivo* and provides a promising therapeutic approach to improve the survival benefits of HCC treatment.

## Supplementary Material

Supplementary methods, figures and tables.

## Figures and Tables

**Table 1:** The IC_50_ of PA and clinical drugs in HCC and normal cells

Cell line	Cell type	PA	SOR	VP-16	Dox	5-FU
HepG_2_	Human HCC cell	78.2 ± 10.3	6.45 ± 2.56	8.29 ± 0.58	8.19 ± 6.4	34.36 ± 11.45
Mahlavu	Human HCC cell	146 ± 10.6	11.68 ± 4.32	8.04 ± 6.07	1.95 ± 1.18	142.68 ± 6.69
J5	Human HCC cell	108.4 ± 18.1	6.58 ± 4.54	20.27 ± 11.04	2.15 ± 0.02	131.61 ± 52.74
Huh7	Human HCC cell	66.5 ± 3.8	2.56 ± 3.25	11.2 ± 9.99	3.26 ± 2.41	54.28 ± 39.9
BNL CL.2	Mouse liver embryonic cell	294.9 ± 7.4	30.83 ± 5.74	7.51 ± 3.84	6.49 ± 2.34	>153.75
SVEC	Mouse vascular endothelial cell	262.8 ± 0.2	23.43 ± 8.26	3.98 ± 1.73	N.D.	23.99 ± 16.76
MDCK	Canine epithelial kidney cell	328.6 ± 1.3	15.04 ± 5.87	20.97 ± 5.25	N.D.	18.22 ± 9.15

Note: Values are the means ± SD (μM) at 48 h. SOR: Sorafenib. VP-16: Etoposide. Dox: Doxorubicin. 5-FU: 5-Fluorouracil.
