# Supplementary material for: Inhibitory effects of patchouli alcohol on hepatocellular carcinoma growth through accumulation of oxidative stress and inactivation of androgen receptor signaling
Source: Int J Med Sci. 2026 Feb 18;23(4):1174–89. doi: 10.7150/ijms.123787 (PMC13048856; doi:10.7150/ijms.123787)
Supplement: Supplementary file 1 — Supplementary methods, figures and tables. [file ijmsv23p1174s1.pdf]

Supplementary Table

Supplementary Table 1. Comparison of selectivity index of different drugs

Normal cells	Tumor cells	PA	SOR	VP-16	Dox	5-FU
**SVEC**	**HepG2**	3.4	3.6	0.5	N.D.	0.7
	**Mahlavu**	1.8	2.0	0.5	N.D.	0.2
	**J5**	2.4	3.6	0.2	N.D.	0.2
	**Huh7**	4.0	9.2	0.4	N.D.	0.4
**MDCK**	**HepG2**	4.2	2.3	2.5	N.D.	0.5
	**Mahlavu**	2.3	1.3	2.6	N.D.	0.1
	**J5**	3.0	2.3	1.0	N.D.	0.1
	**Huh7**	4.9	5.9	1.9	N.D.	0.3
**BNL CL.2**	**HepG2**	3.8	4.8	0.9	0.8	>4.5
	**Mahlavu**	2.0	2.6	0.9	3.3	>1.1
	**J5**	2.7	4.7	0.4	3.0	>1.2
	**Huh7**	4.4	12.0	0.7	2.0	>2.8

Note: Selectivity index (SI) = IC_{50} of normal cells / IC_{50} of HCC cells. SI > 1: indicated that drugs have better selectivity for tumor cells; SI < 1: indicated that drugs have poor selectivity for tumor cells. SOR: Sorafenib. VP-16: Etoposide. Dox: Doxorubicin. 5-FU: 5- Fluorouracil.

Supplementary Table 2. Sequences of primers used for quantitative RT-PCR reactions

Gene name	Forward (5'-3')	Reverse (5'-3')
SNAIL	TCTGAGGCCAAGGATCTCCA	GTGGCTTCGGATGTGCATCT
TWIST	CAGCTACGCCTTCTCGGTCT	CTGTCCATTTTCTCCTTCTCTGG
ICAM-1	GGCCGGCCAGCTTATACAC	TAGACACTTGAGCTCGGGCA
VCAM-1	TCAGATTGGAGACTCAGTCATGT	ACTCCTCACCTTCCCGCTC
MMP9	TATGACATCCTGCAGTGCCC	TTGTATCCGGCAAACTGGCT
KLK3	CCAGACACTCACAGCAAGGA	ATCCCATGCCAAAGGAAGAC
TMPRSS2	GTGATGGTATTCACGGACTGG	CAGCCCCATTGTTTTCTTGTA
GADPH	GAGTCAACGGATTTGGTCGT	GACAAGCTTCCCGTTCTCAG

Supplementary Figure

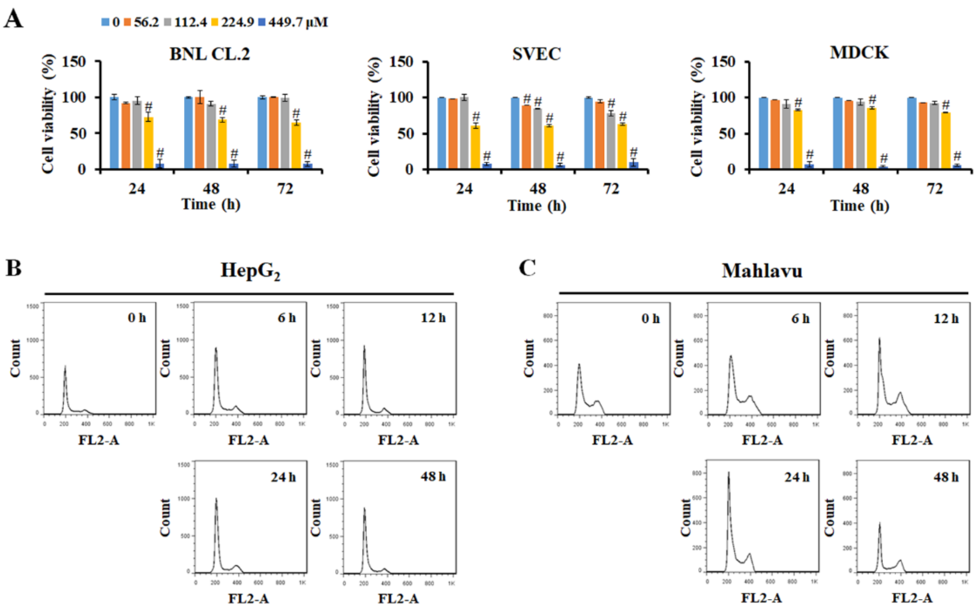

Figure S1. PA-induced cell cycle arrest resulted in growth inhibition of HCC cells. (A) Normal cells (BNL CL.2, SVEC and MDCK) were treated with PA (0–449.7 μM) for 24, 48, and 72 h, and cell viability was measured using MTT assays. $^{\#}P < 0.05$, versus control with significant decrease. (B and C) HepG2 and Mahlavu cells were treated with 89.9 and 112.4 μM PA for 0–48 h, h, stained with propidium iodide and analyzed for FL2-A intensity by flow cytometry.

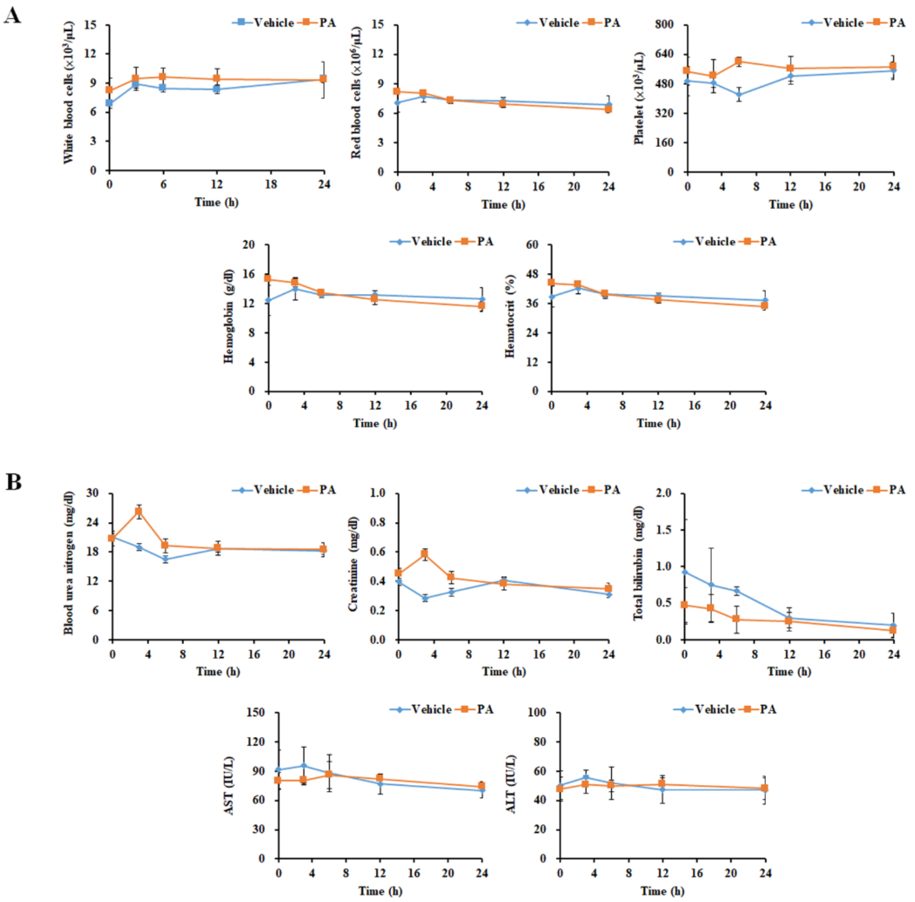

Figure S2. The values of blood cells and serum biochemistry analysis were within normal range before and after PA treatment. Rats were randomly divided into vehicle (n=4) and PA (n=4) groups and treated with one dose of 150 mg/kg PA, and we collected blood samples at 0, 3, 6, 12, and 24 h for blood cells and biochemical analysis. (A) The blood cells were analyzed for white blood cells (WBC), red blood cell (RBC), platelets (PLT), hemoglobin (HGB), and hematocrit (HCT). (B) The serum was analyzed for blood urea nitrogen (BUN), creatinine (CRE), total bilirubin (TBIL), aspartate aminotransferase (AST), and alanine transaminase (ALT). All values in vehicle and PA groups were within normal range, thus suggesting the doses of PA were well-tolerated.

Supplementary methods

Blood cells and serum biochemistry analysis. Rats (8-10 weeks old; F344) were obtained from the National Laboratory Animal Breeding and Research Center (Taipei, Taiwan). Animal experiments were performed at Chung Shan Medical University (CSMU) and approved by the Institutional Animal Care and Use Committee of CSMU (allowance number: CSMU-IACUC-2599). Rats were randomly divided into vehicle (n=4) and PA (n=4) groups and treated with a single dose of 150 mg/kg PA (s.c.). Blood was collected at 0, 3, 6, 12, and 24 h and mixed with EDTA solution for blood cell and biochemical analyses. The values of white blood cell (WBC), red blood cell (RBC), platelets (PLT), hemoglobin (HGB), hematocrit (HCT) in blood cells were analyzed using a hematology analyzer (Sysmex XE-5000), while the levels of blood urea nitrogen (BUN), creatinine (CRE), total bilirubin (TBIL), aspartate aminotransferase (AST), and alanine transaminase (ALT) in serum were analyzed using a biochemistry analyzer (UniCel DxC 800, Beckman Coulter, Inc.).
